# Supplementary material for: Antimicrobial Resistance, Pathogenic, and Molecular Characterization of Escherichia coli from Diarrheal Patients in South Korea
Source: Pathogens. 2022 Mar 23;11(4):385. doi: 10.3390/pathogens11040385 (PMC9030120; doi:10.3390/pathogens11040385)
Supplement: Supplementary file 1 [file pathogens-11-00385-s001.zip › pathogens-1628316-supplementary.pdf]

Supplementary Table S1. O antisera used for determination of *E. coli* serotypes.

Polyvalent sera	Monovalent sera
Polyvalent 1	O1, O26, O86a, O111, O119, O127a, O128
Polyvalent 2	O44, O55, O125, O126, O146, O166
Polyvalent 3	O18, O114, O142, O151, O157, O158
Polyvalent 4	O6, O27, O78, O148, O159, O168
Polyvalent 5	O20, O25, O63, O153, O167
Polyvalent 6	O8, O15, O115, O169
Polyvalent 7	O28ac, O112ac, O124, O136, O144
Polyvalent 8	O29, O143, O152, O164

Supplementary Table S2. Primer pairs used for determination of pathotypes of *E. coli.*

Pathotype	Target gene	Primer Sequence (5′-3′)	PCR products (bp)	References
STEC	*stx1*	CGTACGGGGATGCAGATAAATCGC<br>CAGTCATTACATAAGAACGCCCAC	210	Cho et al., 2006 [1]
	stx2	CTTCGGTATCCTATTCCCGG<br>CTGCTGTGACAGTGACAAAACGC	516	Blanco et al., 2003 [2]
EPEC	*eaeA*	ATGCTGGCATTTGGTCAGGTCGG<br>TGACTCATGCCAGCCGCTGATGCG	233	Cho et al., 2006 [1]
	bfpA	AATGGTGCTTGCGCTTGCTGC<br>GCCGCTTTATCCAACCTGGTA	326	Aranda et al., 2007 [3]
ETEC	*lt*	GATCACGCGAGAGGAACACAAACC<br>ATCTGTAACCATCCTCTGCCGGAG	366	Cho et al., 2006 [1]
	st	CTTTCCCCTCTTTTAGTCAGTC<br>CACAGGCAGGATTACAACAAAGT	167	Cho et al., 2006 [1]
EAEC	*aggR*	CTAATTGTACAATCGATGTA<br>AGAGTCCATCTCTTTGATAAG	457	Moyo et al., 2007 [4]
EIEC	*ial*	GGTATGATGATGATGAGTCCA<br>GGAGGCCAACAATTATTTCC	650	López-Saucedo et al. 2003 [5]

References

1. Cho, S.H.; Kim, J.H.; Kim, J.C.; Shin, H.H.; Kang, Y.H.; Lee, B.K. Surveillance of bacterial pathogens associated with acute diarrheal disease in the Republic of Korea during one year, 2003. *Journal of Microbiology* **2006**, *44*, 327-335.
2. Blanco, M.; Blanco, J.; Mora, A.; Rey, J.; Alonso, J.; Hermoso, M.; Hermoso, J.; Alonso, M.; Dahbi, G.; González, E. Serotypes, virulence genes, and intimin types of Shiga toxin (verotoxin)-producing Escherichia coli isolates from healthy sheep in Spain. *Journal of clinical microbiology* **2003**, *41*, 1351-1356.
3. Aranda, K.R.; Fabbricotti, S.H.; Fagundes-Neto, U.; Scaletsky, I.C. Single multiplex assay to identify simultaneously enteropathogenic, enteroaggregative, enterotoxigenic, enteroinvasive and Shiga toxin-producing Escherichia coli strains in Brazilian children. *FEMS microbiology letters* **2007**, *267*, 145-150.
4. Moyo, S.J.; Maselle, S.Y.; Matee, M.I.; Langeland, N.; Mylvaganam, H. Identification of diarrheagenic Escherichia coli isolated from infants and children in Dar es Salaam, Tanzania. *BMC infectious diseases* **2007**, *7*, 1-7.
5. López-Saucedo, C.; Cerna, J.F.; Villegas-Sepulveda, N.; Thompson, R.; Velazquez, F.R.; Torres, J.; Tarr, P.I.; Estrada-García, T. Single multiplex polymerase chain reaction to detect diverse loci associated with diarrheagenic Escherichia coli. *Emerging infectious diseases* **2003**, *9*, 127.

Supplementary Table S3. Determination of susceptible, intermediate, and resistant bacteria to antimicrobial agents based on the standard set by CLSI.

Antimicrobial agents	Susceptibility	Intermediate	Resistance
Ampicillin (AM)	≥17	14-16	≤13
Amikacin (AN)	≥17	15-16	≤14
Chloramphenicol (C)	≥18	13-17	≤12
Cephalothin (CF)	≥18	15-17	≤14
Ciprofloxacin (CIP)	≥21	16-20	≤15
Cefotetan (CTT)	≥16	13-15	≤12
Cefotaxime (CTX)	≥23	15-22	≤14
Cefazolin (CZ)	≥18	15-17	≤14
Cefepime (FEP)	≥18	15-17	≤14
Gentamicin (GM)	≥15	13-14	≤12
Imipenem (IPM)	≥16	14-15	≤13
Nalidixic acid (NA)	≥19	14-18	≤13
Ampicillin/Sulbactam (SAM)	≥15	12-14	≤11
Trimethoprim/sulfamethoxazole (SXT)	≥16	11-15	≤10
Tetracycline (TE)	≥19	15-18	≤14
Ticarcillin (TIC)	≥20	15-19	≤14-

Supplementary Table **S4.** Pathotyping by virulence gene, O-serotyping, and molecular typing by RAPD and MALDI TOF of *E. coli* isolates.

Strain number	Species	Pathotype	Virulence gene	Serogroup	RAPD	MALDI TOF
EC1	*E. coli*	ETEC, EPEC	*st, bfpA*		V	B
EC2	*E. coli*	EPEC	*eaeA*		III	A
EC3	*E. coli*	EPEC	*eaeA*	O119	II	D
EC4	*E. coli*	EPEC	*eaeA*		III	A
EC5	*E. coli*	ETEC, EPEC	*st, eaeA, bfpA*	O8	II	D
EC6	*E. coli*	ETEC, EPEC	*st, eaeA*		II	B
EC7	*E. coli*	EPEC	*eaeA*	O18	III	D
EC8	*E. coli*	EPEC	*eaeA*		II	D
EC9	*E. coli*	EPEC	*eaeA*	O8	II	D
EC10	*E. coli*	EPEC	*bfpA*		II	C
EC11	*E. coli*	EPEC	*eaeA*	O119	III	C
EC12	*E. coli*	EPEC	*eaeA*		II	D
EC13	*E. coli*	ETEC	*st*	O25	II	D
EC14	*E. coli*	EPEC	*eaeA, bfpA*		III	A
EC15	*E. coli*	ETEC, EPEC	*st, eaeA*		III	A
EC16	*E. coli*	ETEC	*st*	O25	I	A
EC17	*E. coli*	ETEC	*st*	O25	I	A
EC18	*E. coli*	EPEC	*eaeA, bfpA*		V	A
EC21	*E. coli*	EPEC	*eaeA*	O20	II	D
EC22	*E. coli*	EPEC	*eaeA*	O8	II	D
EC24	*E. coli*	ETEC	*st*		II	D
EC25	*E. coli*	ETEC	*st*	O159	II	D
EC26	*E. coli*	EPEC	*bfpA*		II	D
EC27	*E. coli*	EPEC	*eaeA*	O127a	III	B
EC28	*E. coli*	ETEC, EPEC	*st, bfpA*		I	D
EC30	*E. coli*	EPEC	*bfpA*		II	D
EC31	*E. coli*	EPEC	*eaeA*		II	D
EC32	*E. coli*	EPEC	*bfpA*	O6	V	A
EC33	*E. coli*	EPEC	*eaeA*	O166	I	D
EC34	*E. coli*	EPEC	*eaeA*	O166	I	D
EC35	*E. coli*	EPEC	*bfpA*		II	D
EC36	*E. coli*	EPEC	*bfpA*	O153	I	A
EC37	*E. coli*	EPEC	*eaeA*	O125	II	C
EC39	*E. coli*	EPEC	*eaeA*	O119	II	D
EC40	*E. coli*	EPEC	*eaeA*	O18	II	D
EC41	*E. coli*	EPEC, EAEC	*eaeA, aggR*	O159	II	D
EC42	*E. coli*	EPEC	*eaeA*	O55	I	D
EC43	*E. coli*	EPEC	*eaeA*		I	D
EC44	*E. coli*	EPEC	*eaeA*	O20	II	D
EC45	*E. coli*	EPEC	*eaeA*	O127a	II	D
EC46	*E. coli*	ETEC, EAEC	*st, aggR*		II	D
EC47	*E. coli*	EPEC, EAEC	*eaeA, aggR*	O159	III	A

EC48	*E. coli*	EPEC	*eaeA*	O153	II	D
EC49	*E. coli*	EPEC	*eaeA*		II	D
EC50	*E. coli*	EPEC	*eaeA*		III	A
EC51	*E. coli*	EPEC	*eaeA*		II	D
EC52	*E. coli*	EPEC	*eaeA*			A
EC53	*E. coli*	EPEC	*eaeA*	O28ac	III	C
EC54	*E. coli*	EPEC, EAEC	*eaeA, aggR*	O169	IV	D
EC55	*E. coli*	ETEC, EAEC	*st, aggR*			D
EC56	*E. coli*	EAEC	*aggR*	O20	II	D
EC57	*E. coli*	EAEC	*aggR*		II	D
EC58	*E. coli*	EAEC	*aggR*		II	D
EC59	*E. coli*	EAEC	*aggR*	O25	I	D
EC60	*E. coli*	EAEC	*aggR*		II	D
EC61	*E. coli*	EAEC	*aggR*	O166	IV	B
EC62	*E. coli*	EAEC	*aggR*	O1	II	D
EC63	*E. coli*	EAEC	*aggR*		IV	D
EC64	*E. coli*	EAEC	*aggR*	O78	II	D
EC65	*E. coli*	EAEC	*aggR*		II	D
EC66	*E. coli*	EAEC	*aggR*		II	D
EC67	*E. coli*	EAEC	*aggR*	O28ac	II	D
EC68	*E. coli*	EAEC	*aggR*		II	D
EC69	*E. coli*	EAEC	*aggR*		II	D
EC70	*E. coli*	EAEC	*aggR*	O28ac	II	D
EC71	*E. coli*	EAEC	*aggR*		II	D
EC72	*E. coli*	EAEC	*aggR*		II	D
EC73	*E. coli*	EAEC	*aggR*		II	D
EC74	*E. coli*	EAEC	*aggR*	O166	IV	B
EC75	*E. coli*	EAEC	*aggR*	O166	IV	B
EC76	*E. coli*	EAEC	*aggR*	O20	II	D
EC77	*E. coli*	EAEC	*aggR*	O8	II	D
EC78	*E. coli*	EAEC	*aggR*		II	C
EC79	*E. coli*	EAEC	*aggR*	O166	IV	B
EC80	*E. coli*	EAEC	*aggR*		II	D
